# The otolith vermis: A systems neuroscience theory of the Nodulus and Uvula

**DOI:** 10.3389/fnsys.2022.886284

**Published:** 2022-09-15

**Authors:** Jean Laurens

**Affiliations:** Ernst Strüngmann Institute (ESI) for Neuroscience in Cooperation with Max Planck Society, Frankfurt, Germany

**Keywords:** cerebellum, vestibular, Kalman filter, gravity, internal model

## Abstract

The Nodulus and Uvula (NU) (lobules X and IX of the cerebellar vermis) form a prominent center of vestibular information processing. Over decades, fundamental and clinical research on the NU has uncovered many aspects of its function. Those include the resolution of a sensory ambiguity inherent to inertial sensors in the inner ear, the otolith organs; the use of gravity signals to sense head rotations; and the differential processing of self-generated and externally imposed head motion. Here, I review these works in the context of a theoretical framework of information processing called the internal model hypothesis. I propose that the NU implements a forward internal model to predict the activation of the otoliths, and outputs sensory predictions errors to correct internal estimates of self-motion or to drive learning. I show that a Kalman filter based on this framework accounts for various functions of the NU, neurophysiological findings, as well as the clinical consequences of NU lesions. This highlights the role of the NU in processing information from the otoliths and supports its denomination as the “otolith” vermis.

## Introduction

**FIGURE 1:**
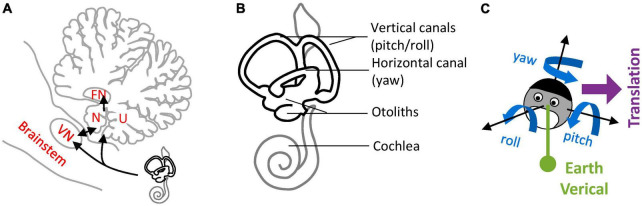
Nodulus and Uvula (NU) and brainstem/cerebellar networks that process 3D head motion. **(A)** Drawing of the NU, represented on a sagittal section of the cerebellum through the midline. The Nodulus (N) and Uvula (U) correspond to the Xth and IXth lobules of the vermis, respectively. The VN and FN are also represented. Connections between the vestibular organs and these regions are shown by black arrows. **(B)** Drawing of the vestibular organs (black) and cochlea (gray) in the inner ear. The vertical and horizontal semicircular canals are sensitive to head rotations in 3D, and the otoliths to tilt and translation. **(C)** Variables used to describe 3D head motion. 3D rotations are decomposed into yaw, pitch and roll rotations, expressed in an egocentric frame of reference. Head tilt is expressed as relative to the allocentric earth vertical. Translational motion is expressed as an egocentric 3D vector.

Lobules IX and X of the cerebellar vermis, also known as the Nodulus and Uvula (NU) (Figure 1A), are a prominent center of vestibular information processing. Over decades of vestibular research, the NU has been studied from many perspectives: anatomical, physiological, clinical, and theoretical. Anatomically, the NU is the recipient of abundant primary and secondary projections (Figure 1A, black) from the vestibular organs (Figure 1B) that sense head motion in 3D (Figure 1C). It also connects with prominent components of the subcortical vestibular network: vestibular nuclei (VN), fastigial nucleus (FN) (Figure 1A), and vestibular regions of the inferior olive (IO) (Bernard, 1987; Voogd and Barmack, 2006; Voogd et al., 2012). Physiologically, recordings of Purkinje cells have shown that they participate in a well-defined central computation that separates gravity and translation head motion from signals from the otoliths (Angelaki et al., 2004; Yakusheva et al., 2007; Laurens et al., 2013b). Clinically, lesions of the NU disrupt the sensing of head rotation by altering a process called velocity storage (VS) (Waespe et al., 1985; Solomon and Cohen, 1994; Angelaki and Hess, 1995a,b; Wearne et al., 1998; Meng et al., 2014). Theoretically, the NU is understood as the neuronal implementation of an internal model of head motion (Merfeld, 1995; Laurens and Angelaki, 2011, 2017; Karmali and Merfeld, 2012).

This multiplicity of viewpoints complicates the effort to understand the role of the NU in vestibular information processing and raises the question of whether the NU performs a unitary function at all. Here, I show that physiological and clinical findings can be explained by a single theoretical concept: that the NU implements an internal model of head motion to predict the activation of the otoliths, and outputs sensory prediction errors that are broadcasted to other brain regions to correct internal estimates of self-motion, or to drive learning. Based on the afferent and efferent connections of the NU, and the physiology of neighboring regions, I discuss the position of this internal model in the anatomical vestibular and cerebellar networks.

This framework indicates that the NU plays a pivotal role in processing information from the otoliths and sends otoliths-based feedback to other brain regions, hence supporting the notion of the NU as the “otolith” vermis.

## Tilt/translation discrimination

**FIGURE 2:**
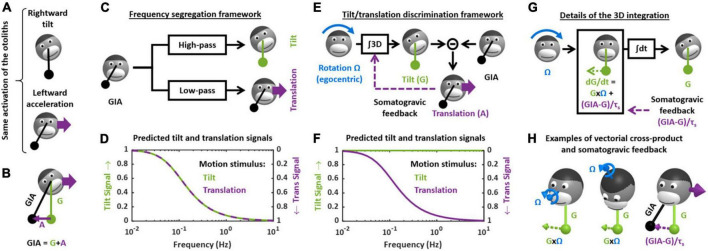
Theoretical frameworks for the resolution of the gravito-inertial ambiguity. **(A)** Illustration of the ambiguity: the otolith organs are analogous to a pendulum (black) that swing relative to the head during tilt (top) or translation (bottom). **(B)** Physical model: the otoliths sense the GIA, which is the sum of gravitational (G) and inertial (A) acceleration. **(C)** Outline of the frequency segregation hypothesis. **(D)** Predicted internal tilt and translation signals during tilt (green) and translational (violet) motion, based on the frequency segregation hypothesis. Any given stimulus (i.e., tilt or translation at a given frequency) is decomposed into a tilt and translation signals. The left and right ordinate axes indicate the amplitude of the tilt and translation signals, respectively, relative to the amplitude of the stimulus. Note that, based on the frequency segregation hypothesis, these internal signals are identical during tilt and translational motion. **(E)** Outline of the internal model hypothesis. **(F)** Predicted internal tilt and translation signals based on the internal model hypothesis. **(G)** Decomposition of the 3D integration in two steps: computing dG/dt as a function of rotation signals (G × Ω) and of the somatogravic feedback [(GIA-G)/τ_*s*_] and temporal integration (∫dt). **(H)** Illustration of the vectorial cross-product (G × Ω) and somatogravic feedback in 3D. The left and middle panels illustrate the cross-product during roll tilt (left) and yaw rotation around a tilted axis (OVAR, middle). The right panel illustrates the somatogravic feedback during leftward acceleration.

Tilt/translation discrimination is one of the fundamental steps of central vestibular information processing. It resolves a sensory ambiguity by disambiguating the sensory signals from the otoliths that cannot discriminate tilt from translation. This is easily illustrated by considering the following analogy: the otolith organs are similar to a pendulum fixed to the head that swings relative to the head during tilt or translation motion (Figure 2A). Thus, based on otoliths signals alone, it is impossible to distinguish tilt from translational motion (Einstein, 1907).

After a brief summary of the theoretical concepts involved in tilt/translation discrimination, I will review the involvement of the NU and associated vestibular networks:

### Theoretical framework

From a physical point of view, the otolith organs sense the gravito-inertial acceleration (GIA), which can be expressed as the sum of gravitational (G) and inertial (A) accelerations (Figure 2B). These two accelerations are in fact physically equivalent (Einstein, 1907), and it is therefore impossible to separate them based on otolith cues alone. In this respect, the otolith organs are inherently ambiguous.

How (or whether) the brain deals with this ambiguity has been the subject of considerable attention and debate, both at the experimental and theoretical levels (Mayne, 1974; Angelaki et al., 1999, 2004; Hess and Angelaki, 1999; Merfeld et al., 1999, 2005a,2005b; Bos and Bles, 2002; Raphan and Cohen, 2002; Laurens and Angelaki, 2011; Yakushin et al., 2017; Jamali et al., 2019). For several decades, two distinct hypotheses existed. The first, called the “frequency segregation” hypothesis (Seidman and Paige, 1996; Raphan and Cohen, 2002), stipulates that the brain does not explicitly distinguish tilt from translation, but separates the low-frequency and high-frequency components of the otolith signals (Figure 2C), and interprets the low-frequency component as tilt and the high-frequency component as translation (Figure 2D). This framework implies that motion sensation should be identical during tilt and translation: indeed, the brain would interpret otolith signals based on their frequency content alone, and not whether the head is really tilting or translating.

The second hypothesis stipulates that the brain uses semicircular canals information to separate tilt from translation. Indeed, tilt movements are rotations and are sensed by the canals. By integrating rotation velocity signals in three dimensions (“∫3D box” in Figure 2E), the brain can compute head tilt relative to gravity (Figure 2E). Once head tilt is known, translation can be computed by a simple subtraction (A = GIA-G, Figure 2E) and the gravito-inertial ambiguity is resolved. This hypothesis is part of a more general framework called the internal model theory, which assumes that the brain uses internal representations of head motion (here tilt and translation) that match sensory signals as well as the physical laws governing head motion (here the causal relationship between rotation and tilt) and the sensory organs (here the physical principle of gravito-inertial ambiguity).

One limitation of this process is that, in the absence of corrective mechanism, the 3D integration would tend to accumulate errors that result from inaccurate rotation signals. To prevent this, most models add a feedback loop that continuously biases the tilt estimate toward the GIA (Figure 2E, “somatogravic feedback”). This feedback mitigates the accumulation of errors by imposing the GIA as a reference for tilt at low frequencies. It also implies that low-frequency translations are interpreted as head tilt (Figure 2F), and in this respect the discrimination model is similar to the frequency segregation model.

The crucial step in the tilt/translation discrimination model is the 3D integration. This step is developed in detail in Figures 2G,H. Mathematically, the 3D integrator computes an estimate of the 3D position of the gravity vector (G) in egocentric coordinates, based on rotation signals (Ω) and on the somatogravic feedback. The somatogravic feedback itself is proportional to the acceleration signal (A = GIA-G) and can be expressed as (GIA-G)/τ_*s*_, where τ_*s*_ is the time constant with which the somatogravic illusion develops during constant linear acceleration. This integration can be divided into two steps. The first step computes how G varies (i.e., dG/dt) based on the rotation signal Ω: this is accomplished by a vectorial cross-product G × Ω. This is illustrated by two examples in Figure 2H (left and right panel): in both cases, the rotation Ω causes the head to tilt toward the right side. Accordingly, the vectorial cross-product G × Ω is a vector that points to the right, indicating that G moves rightward. In addition, dG/dt is computed by adding the somatogravic feedback to G × Ω. As illustrated in the right panel of Figure 2H, this feedback tends to align G toward the GIA. Finally, dG/dt is integrated over time to compute G.

In agreement with both models, experiments in humans and non-human primates have revealed that low-frequency translation is indeed interpreted as head tilt: this effect is called oculogravic or somatogravic illusion (Graybiel, 1952; Graybiel et al., 1979; Paige and Tomko, 1991; Curthoys, 1996). Both the frequency filtering and the discrimination model interpret this effect by pointing out that low-frequency accelerations are very infrequent in everyday’s life. Therefore, if the brain cannot discriminate low-frequency tilt from translation, it is logic to interpret both as tilt. The two models differ upon the reason why the brain cannot discriminate low-frequency tilt from translation. In the discrimination framework, this is because the integration process accumulates error and therefore becomes unreliable at low frequencies (Laurens and Droulez, 2007; Laurens and Angelaki, 2017). In the filtering model, it is because the brain never discriminates them in the first place.

The crucial experiment to distinguish these frameworks is to test whether the brain can discriminate high-frequency tilt from translation, as predicted by the discrimination model. This model also predicts that artificially activating the canals can induce illusory translation. From the last 90s onward, these predictions were both confirmed by a series behavioral studies in macaques (Angelaki et al., 1999; Hess and Angelaki, 1999; Laurens et al., 2010) and humans (Merfeld et al., 1999; Vingerhoets et al., 2007; Khosravi−Hashemi et al., 2019). These behavioral results, which were themselves conclusive, were followed by a series of neurophysiological studies that firmly confirmed the disambiguation model and identified some of its neuronal correlates, as will be discussed next.

### Tilt- and translation-selective neurons

Starting in the early 2000s, a series of studies have uncovered neurons that encode specifically translation or tilt (called translation- and tilt-selective neurons, respectively), thereby providing a direct and compelling confirmation of the discrimination model. These neurons exist in the NU, and in regions closely associated with it, including the fastigial and VN. In the NU, these neurons amount to about two-third of the Purkinje cells and are the only Purkinje cells for which a clearly defined function has been proposed. This suggests that the NU is indeed mainly involved in computation related to tilt/translation discrimination. In this section, I will summarize these experiments and the properties of translation- and tilt-selective cells in the NU.

**FIGURE 3:**
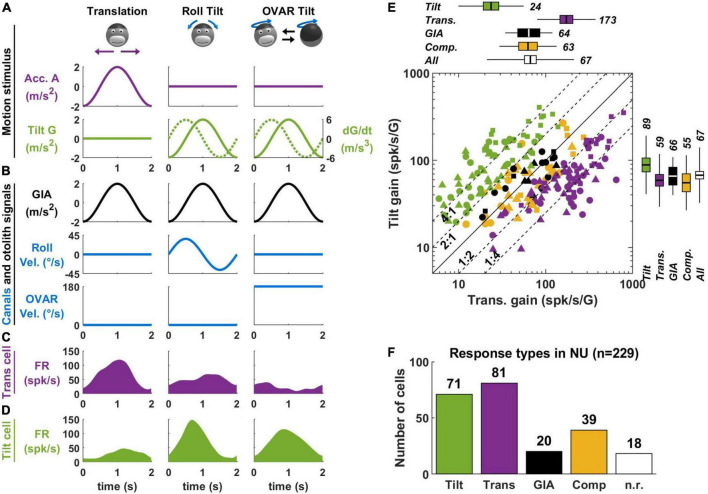
Translation- and tilt-selective neurons in the NU. **(A)** Example motion stimuli used in tilt/translation discrimination experiments. **(B)** Sensory signals during these experiments. Roll and OVAR velocity refer to the head’s rotation velocity about its naso-occipital and vertical axis, which correspond to the rotations illustrated in panel **(A)**. **(C,D)** Firing rate of example translation-and tilt-selective cells during a cycle of rotation. **(E)** Scatterplot of the response gain during tilt and translation across the NU. Tilt-selective cells (green) respond preferentially to tilt compared to translation and appear above the diagonal. Reciprocally, translation-selective cells (violet) appear below the diagonal. Other cell types (GIA-selective, yellow and composite, black) appear near to the diagonal. **(F)** Distribution of responses types across the NU: about a third (71/229) cells are tilt-selective, and about a third (81/229) are translation-selective. Other cell types form the remaining third: note that 18 non-responsive cells (n.r., white) don’t appear in panel **(E)**. Data replotted from Laurens et al. (2013b).

Most experiments on tilt/translation discrimination use an experimental paradigm where the head is translated in the horizontal plane (Figure 3A, translation) or tilted around a horizontal axis (Figure 3A, roll tilt). The motion profiles are matched such that the activation of the otoliths is identical during both paradigms (Figure 3B, GIA, black). Therefore, these motions may only be discriminated on the basis of semicircular canal signals, which are activated during tilt but not translation (Figure 3B, roll velocity, blue). Note that I have illustrated only lateral motion in Figure 3 for simplicity, but that this protocol can be repeated along multiple directions to establish the cell’s spatial tuning. In-depth mathematical analyses of these experiments can be found in Green et al. (2005); Laurens and Angelaki (2016).

In the early 2000s, a series of studies (Angelaki et al., 2004; Shaikh et al., 2005; Yakusheva et al., 2007, 2008, 2010) identified so-called “translation-selective” cells whose firing rate is modulated by translation but much less during tilt (Figure 3C). The existence of these cells was a major conceptual advance, since it was the first physiological demonstration that the brain discriminates tilt from translation.

In a more recent series of studies (Laurens et al., 2013b; Stay et al., 2019; Laurens and Angelaki, 2020), we identified so-called “tilt-selective” neurons whose firing rate is modulated by tilt but much less during translation (Figure 3D). Subsequently (Laurens and Angelaki, 2020), we established that these tilt-selective cells encode an intermediate computation step in the 3D integration (Figure 2E), namely, the computation of dG/dt (Figure 2G). Specifically, we found that they encode both transformed rotation signals, i.e., G × Ω, and the somatogravic feedback [see Laurens and Angelaki (2020) for details].

A crucial element for identifying tilt-selective cells was the use of 3D motion protocols (Figures 3A–D, right column). During roll tilt, the egocentric roll velocity (Figure 3B) and the allocentric velocity dG/dt (Figure 3A, broken line) follow a similar profile: based on this motion alone, we cannot distinguish which is encoded by neurons. To resolve this, we designed an additional tilt protocol where animals rotated at a constant velocity about a tilted axis [off-vertical axis rotation (OVAR)]. This created a periodic tilt stimulus with the same tilt and tilt velocity profiles along the head’s lateral axis compared to roll motion (Figure 3A, green). Critically, the egocentric velocity was different: we used a sinusoidal rotation during roll and a constant-velocity rotation during OVAR (Figure 3B, blue). Therefore, cells that encode egocentric velocity would necessarily respond differently during sinusoidal tilt and OVAR. Instead, we found that tilt-selective cells respond similarly during these motions (Figure 3D), thus confirming that they encode allocentric tilt velocity.

In Laurens et al. (2013b), we determined how many Purkinje cells in the NU of macaques are translation-selective, tilt-selective, or encode other variables. About a third of neurons are translation-selective cells, and about a third are tilt-selective cells (Figures 3E,F). The remaining third did not have significantly different responses during tilt and translation: we classified them as GIA-selective (when their responses to both stimuli were approximately similar) or composite otherwise. Few neurons responded neither to tilt nor to translation (n.r. in Figure 3F). The results of this study were confirmed by independent recordings in our labs, in macaques (Laurens and Angelaki, 2020) and mice (Stay et al., 2019). In addition, one study has found translation-selective neurons in the input layer of the NU, i.e., the granular layer (Meng et al., 2014). Crucially, we tested that tilt- and translation-selective cells conformed to predictions of the tilt/translation discrimination framework in Laurens et al. (2013a,b). Together, these studies provide extensive experimental and theoretical support for the concept of tilt/translation discrimination.

## Vestibular network for tilt/translation discrimination

To date, neurons involved in tilt/translation discrimination have been identified in three interconnected regions: the NU, as described above, the FN, and the VN. In addition, we found that IO neurons that project to tilt- and translation-selective cells in the NU are translation-selective themselves. By combining these findings with anatomical studies, I propose that tilt/translation discrimination occurs in an anatomical network outlined in this section.

### Subregions of the Nodulus and Uvula

**FIGURE 4:**
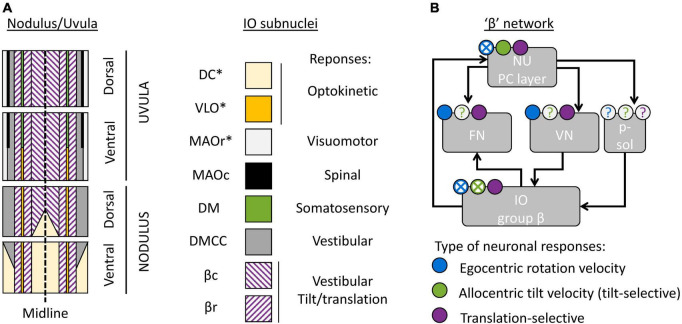
Cerebellar and brainstem nuclei involved in tilt/translation discrimination. **(A)** Schematic map of the NU, indicating the regions innervated by distinct IO subnuclei (Voogd et al., 1996, 2013; Voogd and Barmack, 2006). DC, Dorsal Cap; VLO, ventrolateral outgrowth; MAOr/MAOc, rostral/caudal part of the medial accessory olive; DM, dorsomedial group; DMCC, dorsomedial cell column; βc/βr, caudal/rostral part of the group β. Stars indicate IO subnuclei that project to the flocculus and/or paraflocculus. **(B)** Interconnections between cerebellar and brainstem regions connected to the group β. The circular symbols placed above each region indicate the presence of absence of three types of neuronal response (egocentric rotation velocity, tilt-selective and translation-selective responses are color-coded in blue, green and violet respectively). Filled symbols and crosses indicate, respectively, that the presence or absence of the corresponding response type has been established. Question marks indicate that it is still unknown. p-sol: parasolitary nucleus.

First, the NU is not a homogenous region, but can be divided further into subregions innervated by different subnuclei of the IO. The organization of these subregions has been studied and reviewed in detailed by Voogd et al. (1996, 2012, 2013) in several publications; available data indicate that it is well conserved across model species (rat, rabbits, cats, and likely non-human primates). This organization is outlined in Figure 4A.

The most prominent subregion of the NU is a large medial region innervated by the group β of the IO (Figure 4A, violet diagonal lines). Anatomical reconstructions in Yakusheva et al. (2007; 2010), Laurens et al. (2013b) indicate that tilt- and translation-selective cells are found throughout the NU, in a large region that spans most of the medial portion of the vermis (Figure 4A, violet). Therefore, it is likely that this region coincides with the region innervated by the group β. This conclusion is further supported by other studies that found vestibular responses in this region (Barmack and Shojaku, 1995; Fushiki and Barmack, 1997; Yakhnitsa and Barmack, 2006; Kitama et al., 2014). Although these studies did not investigate tilt/translation discrimination specifically, they found that this region is primarily sensitive to vestibular stimulation. They also further subdivided it into two sagittal bands that preferentially respond to motion along the ipsilateral posterior canal plane (most medially) and ipsilateral anterior canal plane (more laterally). These bands correspond to the caudal and rostral parts of the nucleus β. Note that the dorsal uvula only received sparse projections from the vestibular organs and VN, unlike the rest of the NU (Voogd et al., 1996; Voogd and Barmack, 2006). Yet, there appear to be at least some translation-selective cells in the dorsal uvula (Yakusheva et al., 2007, 2010): current data are insufficient to determine whether these cells are sparser.

What is the function of other subregions of the NU? To date, only a partial answer may be formulated. First, a sizeable portion of the nodulus is innervated by the DC of the IO (Figure 4A, yellow), and a narrow band is innervated by the VLO (Figure 4A, orange). These two regions of the IO are sensitive to optokinetic stimuli, i.e., to retinal flow. It is likely that the Purkinje cells in these regions are more specialized in the processing of visual stimuli. Accordingly, Yakusheva et al. (2013) have shown that a population of NU neurons respond to visual stimulation, that this population is distinct from tilt- and translation-selective cells, and that it is spatially restricted to a subregion of the NU located anterior and medially, which could match the ventral nodulus. Note that the DC and VLO also project to the flocculus and paraflocculus (Voogd et al., 1996), and therefore these regions may be part of a network involved in oculomotricity.

Finally, the most lateral zones of the NU are innervated by the MAO, DM, and DMCC. These regions of the IO receive projections from a variety of systems: vestibular, spinal, somatosensory, and visuomotor. To date, no recording studies have established the function of these regions.

### The “β” network

Anatomical studies have identified subregions of the FN and VN that connect to the group β of the IO or the corresponding regions of the NU. Together, these regions form what may be called a “β” network. I will describe this network here.

First, the NU projects to the ipsilateral FN (Figure 4B). Within the FN, projections from the NU terminate in a ventral subdivision (Armstrong and Schild, 1978; Dietrichs, 1983; Bernard, 1987; Ikeda et al., 1989; Fujita et al., 2020). That subdivision is distinct from the most prominent subdivisions of the FN, which are the “rostral” and “caudal” FN: the “rostral” FN is a relay between the anterior vermis and the spinal VN (Voogd, 2016; Fujita et al., 2020) and the “caudal” FN is an oculomotor subnucleus (Ikeda et al., 1989; Fujita et al., 2020). Projections from the NU terminate in a region located caudally relative to the “rostral” FN and ventrally and somewhat rostral relative to the “caudal” FN. Interestingly, this region may also receive projections from the group β in the IO (Dietrichs and Walberg, 1985). Together, these studies indicate that there is a “β” subnucleus of the FN that likely corresponds to the module F4 described in Fujita et al. (2020).

The NU also projects to the ipsilateral VN (Figure 4B; Bernard, 1987; Xiong and Matsushita, 2000). Note however that the exact location of NU target neurons within the VN has never been firmly established.

Finally, the group β of the IO likely receives indirect projections from the NU. Indeed, it receives projection from the VN (Barmack et al., 1993; Balaban and Beryozkin, 1994). Alternatively, the NU may project to the group β through the parasolitary nucleus (Figure 4B), which is an anatomical relay between these regions (Barmack et al., 1993, 1998; Balaban and Beryozkin, 1994; Barmack and Yakhnitsa, 2000).

### Tilt/translation discrimination through the “β” network

In addition to the NU, several studies have identified neuronal correlates of tilt/translation discrimination through the “β” network.

First, translation-selective neurons have been found in the FN of macaque monkeys (Angelaki et al., 2004; Shaikh et al., 2005; Laurens and Angelaki, 2016; Mackrous et al., 2019). However, there is some uncertainty regarding the exact location of these recordings in respect to NU projections. Although these studies reported that their recordings occurred in the “rostral” FN, they did not perform histological reconstruction. Therefore, they likely could not locate their recordings with enough precision to distinguish between the “rostral” and “β” portions of the FN. Note that (Mackrous et al., 2019) found that a third of neurons are potentially tilt-selective neurons. However, they did not perform recordings during 3D motion (as in Figures 3A–D) and it is, therefore, uncertain whether these neurons encode allocentric tilt, as opposed to egocentric rotations.

Translation-selective cells have been identified in the VN (Angelaki et al., 2004; Meng et al., 2014; Mackrous et al., 2019). Importantly, (Meng et al., 2014) recorded 26 VN cells that were targeted by NU projections (and were not eye movement related), and demonstrated that 11 of them were translation-selective, and the rest GIA-selective. Therefore, at least a part of the translation-selective cells in the VN may be targeted by NU projections. However, more detailed studies will be necessary to establish the exact nature and functions of the interconnections between VN and NU.

## The velocity storage

As we saw in the previous section, neuronal recording studies indicate that tilt/translation discrimination is a prominent function of the NU. Yet, lesion studies in monkeys (Waespe et al., 1985; Angelaki and Hess, 1995a,b; Wearne et al., 1998) and humans (Hain et al., 1988; Lee et al., 2017), or electric stimulation studies in monkeys (Solomon and Cohen, 1994; Meng et al., 2014) have linked it to a seemingly unrelated function: the control of a phenomenon called VS.

**FIGURE 5:**
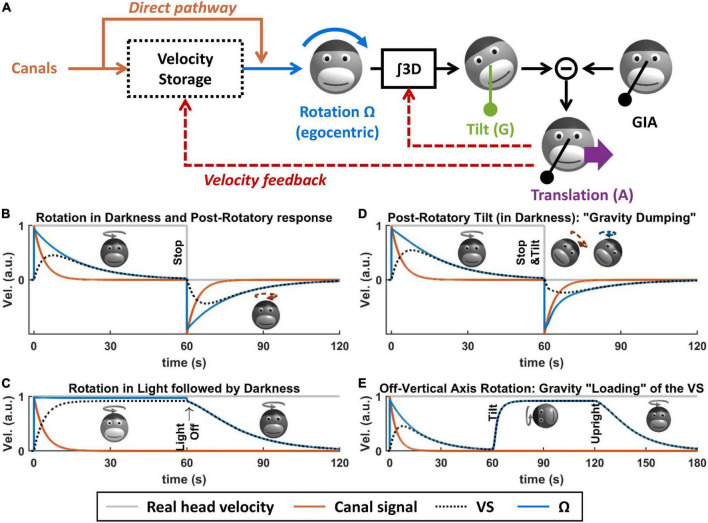
Velocity storage. **(A)** Model of the velocity storage during 3D motion (Laurens and Angelaki, 2011). See text for explanations. **(B–E)** Simulations of rotation perception during rotations in darkness or light, and 3D rotations. Head motion is illustrated by monkey heads, drawn in darker shades when the rotation occurs in darkness. The orange broken arrows in panels **(B,D)** represent the post-rotatory canal activation. The blue broken arrows in panel **(D)** represent the post-rotatory rotation signal.

What is the VS? Based on Bayesian modeling theory, it is the central element of a multisensory internal model that senses head rotation velocity optimally (Laurens and Droulez, 2007; Laurens and Angelaki, 2017). In the context of tilt/translation discrimination, it provides the egocentric rotation velocity signal Ω to the 3D integrator. Prior to this definition, the concept of VS originated in the 70s (Raphan et al., 1979), as a leaky integrator connected to the semicircular canals (Figure 5A, blue and orange). In Laurens and Angelaki (2011), we demonstrated how the VS could be connected to the internal model of tilt/translation discrimination to create a full 3D model of vestibular information processing, as shown in Figure 5A. In Laurens and Angelaki (2017), we demonstrated that the historic model by Raphan and Cohen (Raphan et al., 1979) is equivalent to an optimal Kalman filter.

### Velocity storage during rotations in a horizontal plane

When rotating in complete darkness (and in a horizontal plane), head motion is sensed by the semicircular canals. These canals act as a high-pass filter, with a time constant of ∼4 s. This implies that, during a constant-velocity rotation (Figure 5B, gray), their signal will vanish in about 15–20 s (Figure 5B, orange). Yet, the brain’s sense of rotation will persist for a longer duration, with a time constant of 10–30 s (Raphan et al., 1979; Bertolini et al., 2010; Laurens et al., 2010; Figure 5B, blue). This indicates that a central mechanism increases the time constant of rotation sensation compared to the canals. In their textbook model, Raphan et al. (1979), Raphan and Cohen modeled this mechanism as a leaky integrator (Figure 5B, black) whose output sums with the canals (Raphan et al., 1979), and this integrator was named VS.

Although the VS increases its time constant, rotation sensation keep high-pass characteristics. A consequence of this is that, when stopping after a long period of constant velocity rotation, one experiences an after-effect that is symmetric to the response to the initial rotation (Figure 5B, after *t* = 60 s). This after-effect is the basis of some experimental protocols discussed in the next section.

When rotating relative to a visual surround, rotation sensation persists indefinitely [Figure 5C, note that visual pathways are not shown in Figure 5A for simplicity; see Raphan et al. (1979), Laurens and Angelaki (2011, 2017) for details]. If light is extinguished (without altering the subject’s rotation), then rotation sensation does not cease immediately but decrease exponentially with the same constant during rotation in darkness. This indicates that the VS also store a signal that originates from the visual system.

### Velocity storage during 3D rotations

The previous section described the principles of how the brain processes rotations in a horizontal plane. These principles also apply to rotations in a vertical plane. However, rotating in a vertical plane involves another fundamental mechanism: the interactions between rotation sensation and the otoliths. It is the case because integrating rotation movement is a fundamental part of tilt/translation discrimination, as described above. It is also the case because, reciprocally, otolith signals participate to rotation sensing, as described next.

First, rotation signals can be put in conflict with gravity sensing by the otoliths. A classical paradigm called post-rotatory tilt consists of rotating a subject in darkness, stopping the rotation (as in Figure 5B), and then tilting the subject (Figure 5D, at *t* = 60 s) (Benson, 1974; DiZio and Lackner, 1988; Merfeld et al., 1993, 1999; Angelaki and Hess, 1994, 1995b; Furman and Koizuka, 1994; Gizzi et al., 1994; Fetter et al., 1996; Zupan et al., 2000; Yasuda et al., 2002, 2003; Kitama et al., 2004; Fushiki et al., 2006; Laurens et al., 2010). In an egocentric reference frame, the post-rotatory activity of the canals (Figure 5D, broken arrow) is identical as in a Figure 5B. However, this activity now indicates that the head rotates about a tilted axis. According the internal model framework, this signal is integrated into an estimate of head tilt that varies continuously. However, this estimate will not match the activity of the otoliths since the head is in fact immobile. This mismatch can be resolved by assuming that the head is translating, as has been shown in Merfeld et al. (1999), Laurens et al. (2013a), Khosravi−Hashemi et al. (2019): this will be discussed further in the next section. In addition to this, this mismatch is resolved by altering the central rotation signal (Ω) in two ways. First, its amplitude and duration are reduced (Figure 5D, compare with Figure 5B): this phenomenon is called “gravity dumping.” Second, after the head is tilted, the axis of the rotation signal gradually shifts spatially until it aligns with earth-vertical. This axis shift occurs centrally: the post-rotatory rotation signal generated by the canals remains head-fixed (this is illustrated by a schematic head in Figure 5D, with an orange arrow), but the rotation signal contributed by the VS aligns with earth-vertical (schematic head in Figure 5D, with a blue arrow). Gravity dumping and the realignment of the post-rotatory response with gravity have been observed in several species: squirrel monkeys and macaques (Dai et al., 1991; Merfeld et al., 1993; Angelaki and Hess, 1994, 1995b), cats (Yasuda et al., 2002, 2003; Kitama et al., 2004; Fushiki et al., 2006), and humans (Benson, 1974; DiZio and Lackner, 1988; Furman and Koizuka, 1994; Gizzi et al., 1994; Fetter et al., 1996; Merfeld et al., 1999; Zupan et al., 2000): note that gravity dumping and axis realignment are weaker in humans compared to monkeys. Note that post-rotatory responses align with allocentric vertical even when the initial rotation did not occur about a vertical axis (Dai et al., 1991; Jaggi-Schwarz et al., 2000): this rules out the hypothesis that the axis re-alignment is due to a mechanism that encodes rotation in allocentric coordinates and favors the interpretation that it is a conflict resolution mechanism.

Gravity can also be used to sense head rotation. For instance, when rotating about an earth-horizontal axis in darkness, rotation perception and VOR can last indefinitely (Correia and Guedry, 1966; Harris, 1987; Angelaki and Hess, 1996; Angelaki et al., 2000; Kushiro et al., 2002; Laurens et al., 2010). This can be revealed by first rotating around a vertical axis until rotation sensation subsides (Figures 5E, *t* < 60 s) and then tilting the head while maintaining the rotation (Figure 5E). In this situation, the rotation sensation rapidly resumes (Figure 5E, after *t* = 60 s) and stabilizes to a steady-state level called “bias velocity.” This rotation sensation is mediated by the VS: this can be shown by re-aligning the head with vertical (Figure 5E, at *t* = 120 s). After this, rotation sensation persists and decreases with the typical time constant of the VS (Jaggi-Schwarz et al., 2000; Laurens et al., 2010), indicating that the “bias velocity” signal (until *t* = 120 s) is stored in the VS. The bias velocity can be observed in macaques (Angelaki and Hess, 1996; Angelaki et al., 2000; Kushiro et al., 2002; Laurens et al., 2010), cats (Harris, 1987), and humans (Benson and Bodin, 1966; Correia and Guedry, 1966; Wall and Furman, 1990). Note that the bias velocity varies as a function of tilt angle and saturates or vanishes at high rotation speed (Angelaki et al., 2000; Kushiro et al., 2002; Laurens et al., 2010). Similar to the dumping effect during otolith conflicts, the bias velocity is lower in humans compared to monkeys.

In Laurens and Angelaki (2011), we demonstrated that these results can be explained by the internal model framework, and specifically by a feedback loop from the internal model of tilt/translation discrimination to the VS (Figure 5A, velocity feedback). This will be shown in more detail in the next section.

### Velocity storage and Nodulus and Uvula

From a theoretical point of view, the VS and the general framework of the internal model are well understood. But how are they related to the NU? To date, the neuronal substrate of the VS is unknown. However, lesion studies have shown that the NU is involved in VS in at least two respects. First, NU lesions abolish the influence of gravity on the VS, both in experimental (Waespe et al., 1985; Angelaki and Hess, 1995a,b; Wearne et al., 1998) and in clinical cases (Hain et al., 1988; Lee et al., 2017). Second, NU lesions also alter the time constant of the VS during rotations in a horizontal plane (Waespe et al., 1985; Angelaki and Hess, 1995a,b; Wearne et al., 1998).

Thus, there appears to be a discrepancy between electrophysiological studies, that point to tilt/translation discrimination as the most obvious function of the NU, and lesions studies that suggest that it is involved in the VS. Furthermore, although the VS computes egocentric rotation signals, NU neurons do not encode egocentric rotation velocity (Fushiki and Barmack, 1997; Kitama et al., 2014), which seems to increase the contradiction between these two putative functions. We will see that theoretical models readily provide an answer to this paradox.

## A theoretical framework for Nodulus and Uvula function

I will now propose a theoretical model that conciliates these seemingly dissimilar functions of the NU. This model is grounded in the concept of internal model that was initially proposed in the early 80s (Mayne, 1974; Ormsby and Young, 1977; Oman, 1982; Borah et al., 1988; Young, 2011; Clark et al., 2019) and has evolved into complete 3D models of vestibular information processing (Merfeld, 1995; Bos and Bles, 2002; Laurens and Droulez, 2007; Laurens and Angelaki, 2011, 2017; Karmali and Merfeld, 2012).

### Internal model framework

**FIGURE 6:**
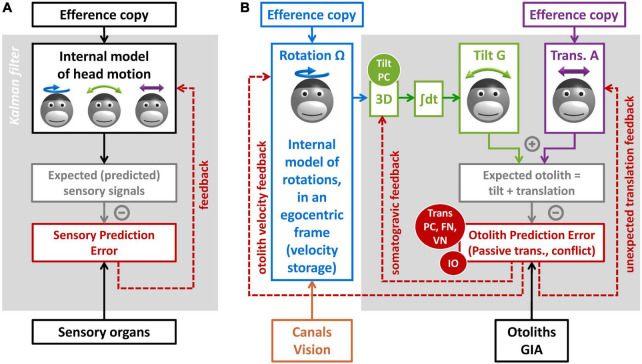
Kalman filter models of vestibular information processing. **(A)** Overview of the Kalman filter algorithm. **(B)** Detail of the Kalman filter model in Laurens and Angelaki (2017), focusing on the processing of otolith information.

The concept of internal model is largely related to the technique of Kalman filtering used in aerospace (Kalman, 1960; Kalman and Bucy, 1961; Figure 6A). It posits that the brain maintains and updates an internal representation of head motion (Figure 6A, black) by two mechanisms. The first is by integrating motor efference copies that encode how the head is expected to move based on voluntary motor activity. The second is a forward model where the brain simulates the sensory organs to anticipate vestibular (and other) sensory efferences (Figure 6A, gray). Any discrepancy between the anticipated and received sensory signals leads to a sensory prediction error (Figure 6A, red), which updates the internal representation of head motion through feedback loops. This framework has received considerable support from its ability to account for the results of behavioral studies (Merfeld et al., 1993, 1999; Glasauer and Merfeld, 1997; Bos and Bles, 2002; Laurens et al., 2010; Laurens and Angelaki, 2011, 2017; Karmali and Merfeld, 2012). It is also supported by neurophysiological findings, as will be described in the next section.

### Internal model for otolith information processing

I will now explain how the internal model framework can explain the multiple functions of the NU. I will use the Kalman filter model in Laurens and Angelaki (2017), and specifically the part of that model dedicated to processing otolith signals (Figure 6B).

The model includes three motion variables that are central to vestibular information processing: (1) the angular velocity of the head in egocentric coordinates (Ω, blue), the allocentric tilt of the head (G, green), and the linear acceleration (A, violet). Note that head rotation and tilt are linked by a causal relationship: changes in head tilt occur through rotation movements, and therefore head tilt is the 3D integral of Ω. These motion variables and the causal relationship between them constitute the internal representation of head motion.

This internal representation can be updated by motor efference copies. Note that this model does not address motor control explicitly. For simplicity, it is assumed that motor centers provide signals that encode self-generated rotations (Figure 6B, blue) and translations (Figure 6B, violet). Note also that the model does not include a distinct efference copy to encode tilt: this is because tilt movements result from rotations; therefore, self-generated tilt is encoded indirectly by the rotation efference copy.

Next, the model computes sensory prediction and sensory prediction error. From a physical point of view, otoliths sense the sum of tilt and translation (Figure 2B). The model mimicks this by summing the internal tilt and translation signals (Figure 6B, gray box). This prediction is subtracted from the actual signal from the otolith reafference (GIA, black), and the result of this subtraction is the otolith prediction error (Figure 6B, red).

### Otolith prediction errors

The concept of otolith prediction error is central to understanding the NU. To fully apprehend it, we may examine when these errors occur or not:

Otolith prediction errors occur during passive or unexpected translations. This is because translations activate the otoliths, but the model does not have any information to anticipate this activation.

Otolith prediction errors occur during canal/otolith conflicts similar to the post-rotatory tilt (Figure 5D). This is because, based on rotation signals (Ω), the model anticipates that the head rotates relative to gravity, but the head is in fact immobile. Multiple experimental paradigms induce canal/otolith conflicts: post-rotatory tilt (Benson, 1974; DiZio and Lackner, 1988; Dai et al., 1991; Merfeld et al., 1993, 1999; Angelaki and Hess, 1994, 1995b; Jaggi-Schwarz et al., 2000; Zupan et al., 2000; Fushiki et al., 2006; Laurens et al., 2010), tilt movement while rotating (Young, 1971; Dichgans and Brandt, 1973; Guedry and Benson, 1976; Bles, 1998; Dai et al., 2009; Laurens et al., 2013a) and direct stimulation of the canals (Khosravi−Hashemi et al., 2019).

In contrast, otolith prediction errors do not occur during active tilt or translations, because motor efference copies allow anticipating the activation of the otoliths.

Based on (Laurens and Angelaki, 2017), otolith prediction errors do not occur during passive tilt either. This is because passive tilt movement is accomplished by passively rotating the head, which induces canal prediction errors. These prediction errors are taken into account by the internal model of rotation (Figure 6B, blue) upstream of the 3D integrator (Figure 6B, green). As a consequence, the internal estimate of tilt (G in Figure 6B) is accurate during passive tilt, and otolith prediction errors do not occur.

Finally, otolith prediction errors can occur during OVAR, but only when the rotation signal Ω does not match the actual rotation of the head. This occurs, for instance, during the first second following head tilt in Figure 5E (Laurens et al., 2011). It also occurs during long-duration, high velocity OVAR, where the VS is not sufficient to maintain an accurate rotation estimate (Laurens et al., 2013b).

### Feedback loops

Next, sensory prediction errors drive feedback loops that update the internal model of head motion. The optimal organization of these feedback loops can be predicted using the Kalman filter algorithm (Kalman, 1960; Kalman and Bucy, 1961). As a rule, each sensory prediction error should drive a feedback loop to update each motion variable. Therefore, in the simplified model of Figure 6B, the otolith prediction error drives feedback loops to the three motion variables: rotation, tilt, and translation. I will discuss each of these loops.

The feedback to the internal model of translation has gain of 1, and therefore that otolith prediction error is interpreted by the brain as an unexpected translation. This allows the internal model to detect passive translations, during which otolith prediction errors occur. This feedback also implies that canal/otolith conflicts should induce a sense of translation: this important prediction was verified by behavioral experiments in Merfeld et al. (1999), Laurens et al. (2013a), Khosravi−Hashemi et al. (2019) and historically provides an important support to the internal model framework. Finally, a sensation of translation should occur during OVAR when Ω does not match the rotation of the head: this has been verified in Vingerhoets et al. (2007), Laurens et al. (2011).

The feedback to the internal model of tilt is fed to the 3D integrator. As a consequence, this feedback matches exactly the somatogravic feedback in Figure 2E. During passive translations, the consequence of this feedback is the somatogravic effect (see Section “Tilt/translation discrimination”). During canal/otolith conflict or during OVAR, this feedback acts to correct the incorrect tilt signals and to decrease the conflict (Laurens et al., 2013a).

Finally, the Kalman filter predicts that the feedback to the rotation estimate is fed to the VS and that it acts to adjust the internal model of rotation to match head motion relative to gravity [see Laurens and Angelaki (2011, 2017)]. As a consequence, this feedback is responsible for the “dumping” (Figure 5D) and realignment of the rotation signal during post-rotatory tilt, and it is also responsible for creating a velocity signal in the VS during OVAR (Figure 5E).

Note that the models in Figures 2E, 5A are included in the model in Figure 6B: the model in Figure 2E is implemented by the 3D integrator and the feedback loops to the internal model of translation and tilt, and the model in Figure 5A is implemented by adding the feedback loop to the internal model of rotation.

This shows that a wide range of behavioral observations can be explained by a simple mechanism where otolith prediction errors drive corrective feedback loops to the internal model of motion. Next, we will discuss how these loops correspond to neuronal response in the central vestibular network. Before this, we can emphasize an important point: the notions of “translation signal” and “otolith prediction error” are closely associated and practically indistinguishable during passive motion, since otolith prediction errors are always interpreted as passive translations.

## The Nodulus and Uvula as a forward model of the otoliths

I will now examine how the internal model frameworks match neuronal responses in the NU and associated regions.

First and foremost, the response of translation-selective cells in the NU and downstream (VN and FN) corresponds precisely to otolith prediction errors. Indeed, by definition, translation-selective neurons in the NU, VN, and FN respond during passive translations but not passive tilt. Furthermore, translation-selective neurons in the NU respond to canal/otolith conflict (Laurens et al., 2013a), or during OVAR when Ω does not match head motion (Laurens et al., 2013b). Finally, translation-selective cells in the FN respond less to self-generated translation compared to passive translations (Mackrous et al., 2019). Based on this, we can propose that these cells, so far described as “translation-selective,” actually encode otolith prediction errors (red oval panel in Figure 6B) that drive the three feedback loops in Figure 6B.

We have determined in Laurens et al. (2013b), Laurens and Angelaki (2020) that tilt-selective cells encode an allocentric tilt velocity signal (see Section “Tilt- and translation-selective neurons”). Accordingly, we propose that they perform the spatial transformation from egocentric rotation signals (Ω) into tilt velocity, which is represented by the block “3D” in Figure 6B. This implies that tilt-selective cells should encode the somatogravic feedback during low-frequency translation: we confirmed this in Laurens et al. (2013a).

Thus, the internal model hypothesis accounts for all known responses in the NU, as well as in the associated regions (Figure 4B). At the same time, it accounts for the consequences of NU lesions on the VS. Indeed, this framework predicts that gravity influences the VS (Figures 2A,D,E) through that velocity feedback loop (Figures 5A, 6B) that originates from the very computations that discriminate tilt from translation. Therefore, NU lesions would automatically abolish the gravity dependence of the VS (Waespe et al., 1985; Hain et al., 1988; Angelaki and Hess, 1995a,b; Wearne et al., 1998; Lee et al., 2017). Furthermore, by eliminating the tonic input from the NU onto neuronal networks that underlie the VS, NU lesions or stimulations may alter its time constant during rotations in a horizontal plane (Waespe et al., 1985; Solomon and Cohen, 1994; Angelaki and Hess, 1995a,b; Wearne et al., 1998; Meng et al., 2014). Note that the neuronal substrate of the VS is yet unknown.

Interestingly, this framework suggests that the velocity feedback to the VS may be driven by translation-selective neurons themselves. This illustrates that the functions of these neurons may be much wider than simply conveying translation signals. In fact, they may be seen as an output channel of the NU that broadcast feedback signals to regulate multiple variables of the internal model.

In the Kalman filter framework, the function of feedback loops is to correct the internal model of motion online. However, our recent finding that IO neurons project to the NU are translation-selective (Angelaki and Laurens, 2021) indicates that IO activity may also encode otolith prediction errors (Figure 6B). Since IO has been involved in cerebellar learning (Lisberger, 1988; Gao et al., 2012), this would point to an additional role where sensory prediction errors are used to control the learning of internal models in the cerebellum.

## Conclusion

Through this review, I have summarized a variety of findings regarding the physiology and function of the NU. I have proposed that these findings are explained by the theory that the NU implements a forward internal model of head motion to predict how the otolith organs are activated by movements on the head and broadcasts feedback signals to other brain regions when prediction errors occur. Note that previous theoretical works based on internal model (Merfeld, 1995; Glasauer and Merfeld, 1997; Bos and Bles, 2002; Laurens and Droulez, 2007; Laurens and Angelaki, 2011, 2017; Karmali and Merfeld, 2012) proposed all-encompassing theories of how multiple motion variables are computing by the brain. The present work does not conflict with these models, but stresses out that the NU implements a forward model of one sensory organ, the otolith, and therefore pinpoints the function of the NU with a greater degree of specificity. Notably, this framework accounts for the NU’s involvement in tilt/translation discrimination, for physiological studies of the NU, and for the NU influence on the VS. On this basis, I propose that the NU may be seen as a section of the vermis dedicated to the otolith organs, i.e., an “otolith vermis.”

The theoretical concept of internal model was initially proposed in the 70s (Mayne, 1974; Ormsby and Young, 1977; Oman, 1982; Borah et al., 1988; Young, 2011; Clark et al., 2019) and evolved into detailed models of vestibular information processing (Merfeld, 1995; Glasauer and Merfeld, 1997; Bos and Bles, 2002; Laurens and Droulez, 2007; Laurens and Angelaki, 2011, 2017; Karmali and Merfeld, 2012). Over the years, it gained strong support based on its ability to explain self-motion perception (Merfeld et al., 1993, 1999; Bos and Bles, 2002; Laurens et al., 2010; Laurens and Angelaki, 2011) and physiological recordings (Angelaki et al., 2004; Cullen, 2012; Laurens et al., 2013a,b). This concept has also gained a wide acceptance in the larger field of motor control (Wolpert et al., 1995; Körding and Wolpert, 2004; Todorov, 2004; Chen-Harris et al., 2008; Sağlam et al., 2014). Here, I have shown that it can account for multiple functions of the NU. This illustrates the predictive power of the internal model framework and supports its use as a normative approach for understanding vestibular function.

It is also worth noting that theoretical models of vestibular information processing were developed and refined based on decades of behavioral data (Merfeld, 1995; Glasauer and Merfeld, 1997; Bos and Bles, 2002; Laurens and Droulez, 2007; Laurens and Angelaki, 2011, 2017; Karmali and Merfeld, 2012). The fact that we now can now identify the neuronal correlates of postulated brain computations illustrates the importance of grounding systems neuroscience in mathematical models of behavior. In this respect, the vestibular system is a unique field that may pioneer the way for studying the principles of sensory-motor control and cerebellar computations.

Earlier modeling works (Merfeld, 1995; Bos and Bles, 2002; Laurens and Droulez, 2007; Laurens and Angelaki, 2011) emphasized how central vestibular computations transform vestibular signals into final estimates of self-motion. In contrast, the Kalman filter framework stresses that self-motion perception is primarily driven by motor efference copies, and how the vestibular organs are primarily used as an error detector, and to generate corrective feedback (Cullen, 2012; Laurens and Angelaki, 2017). This change of viewpoint, largely driven by a series of work in Kathleen Cullen’s laboratory (Cullen, 2012, 2019), is a very significant progress in understanding the vestibular system.

The Kalman filter framework also encourages us to understand the vestibular system in the wider context of motor loops (Wolpert et al., 1995; Körding and Wolpert, 2004; Todorov, 2004; Chen-Harris et al., 2008; Sağlam et al., 2014). By emphasizing the feedback role of the vestibular system, it explains why postural disturbances following vestibular lesions are particularly severe when walking or standing on unstable support that amplify motor errors (McCall and Yates, 2011; Strupp et al., 2016; Sprenger et al., 2017). The optimal estimation framework from which the Kalman filter originates also have the ability to model the consequences of vestibular lesions and sensory substitution (Laurens and Droulez, 2008; Alberts et al., 2018; Angelaki and Laurens, 2020), and is a promising way to study vestibular deficits or vestibular prosthetics.

Recordings in the PC layer of the NU have allowed discovering two components of the internal model highlighted in Figure 6B: the translation- and tilt-selective neurons. In contrast, some crucial components remain to be identified. In particular, neurons encoding the internal model of tilt (G) and the predicted otolith signals remain to be discovered. Furthermore, the neuronal mechanism that conveys motor efference copies to the NU is also unknown. Their discovery and characterization will probably be an exciting challenge for vestibular system neuroscience in the following years. Another challenge will be to understand the multiple functions of vestibular feedbacks in ecological conditions, in health and disease.

## Data availability statement

The original contributions presented in this study are included in the article/supplementary material, further inquiries can be directed to the corresponding author.

## Author contributions

All authors listed have made a substantial, direct, and intellectual contribution to the work, and approved it for publication.
